# Rare and Challenging Tumor Entity: Phyllodes Tumor of the Prostate

**DOI:** 10.1155/2009/241270

**Published:** 2009-12-22

**Authors:** Andreas Bannowsky, Andreas Probst, Helmut Dunker, Tillmann Loch

**Affiliations:** ^1^Department of Urology, Ev.-Luth. Diakonissen Hospital Flensburg, 24939 Flensburg, Germany; ^2^Department of Urology, Klinikum Osnabrück, 49076 Osnabrück, Germany; ^3^Department of Pathology, Ev.-Luth. Diakonissen Hospital Flensburg, 24939 Flensburg, Germany

## Abstract

Cystic epithelial-stromal tumors of the prostate are rare, with 82 cases reported in literature. These cases have been published under a variety of diagnoses, including phyllodes tumor and prostatic stromal proliferation of uncertain malignant potential as well as a malignant tumor called “prostatic stromal sarcoma”. We report a case of a 60-year-old man with the histological diagnosis of phyllodes tumor of the prostate in transurethral resection specimen.

## 1. Introduction

Prostatic cystic epithelial-stromal tumors, commonly described as phyllodes tumors of the prostate, are extremely rare lesions. They exhibit a spectrum of histological features similar to their better-known counterpart in the breast. Although a benign clinical course has been emphasized in some reports, cumulative evidence in literature indicates that these lesions should be considered neoplasms rather than atypical hyperplasia due to the frequent early recurrences with possible dedifferentiation, infiltrative growth, and potential for extraprostatic spread in some cases. An individualized approach to complete excision of the tumor is needed. A case report of a patient recently treated in our hospital is presented, and a review of literature was done.

## 2. Case Report

A 60-year-old man, who visited our hospital, complained of dysuria and bladder outlet obstruction. Digital rectal examination revealed a slightly enlarged benign-feeling prostate. Prostate-specific antigen was 1.1 ng/mL. The urine culture showed no urinary tract infection. X-ray of the kidneys, ureters, and bladder was normal, as was renal ultrasound. Transrectal ultrasound revealed an increased prostate volume (60 mL) without suspicious hypodense or hyperdense areas. Cystoscopy demonstrated enlarged prostate adenoma causing bladder outlet obstruction, which was subsequently resected.

**Figure 1:**
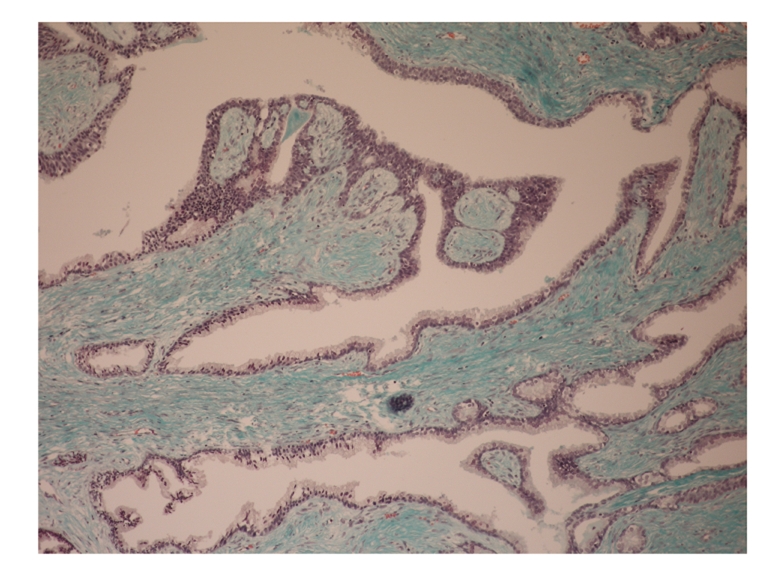
Phyllodes tumor of the prostate, with leaflike protrusions into a cystic cavity and broad stromal compound (Goldner ×40).

**Figure 2:**
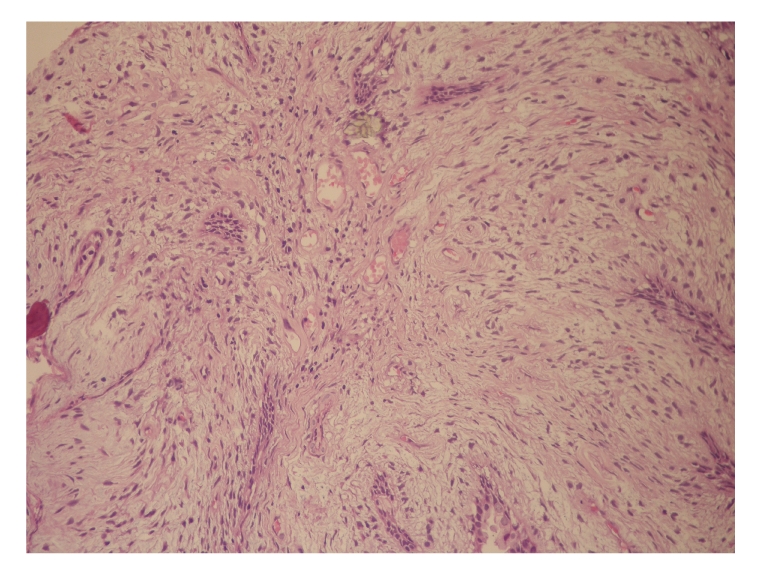
Phyllodes tumor of the prostate: proliferations of elongated and spindle-shaped cells within the stroma and benign glandular elements (H & E ×100).

Pathological examination showed a benign prostatic hyperplasia and features of complex glandular architecture with a prominent stromal component (Figure 1). The stroma consisted of proliferations of elongated and spindle-shaped cells without cytologic atypia or increased mitotic activity (Figure 2). Immunostaining for desmin (smooth muscle marker) and CD34 (mesenchymal marker) confirmed the diagnosis of a phyllodes tumor of the prostate. Stromal cells displayed also immunoreactivity for vimentin but moderate reactivity for actin. Luminal epithelial cells showed intense immunoreactivity for prostate-specific antigen. Due to the absence of necrosis and cellular atypia, this tumor was classified as low-grade “indolent” tumor. 

Additional staging investigations including X-ray of the lung and abdominal magnetic resonance imaging revealed no extracapsular extension of the tumor with absence of lymph node involvement or metastasis. Sextant biopsies of the peripheral zone demonstrated normal prostatic tissue. The patient denied radical surgery, and we performed a second transurethral resection of the prostate three months later without further recurrence of the phyllodes tumor. Due to the malignant potential and the possibility of sarcomatoid differentiation of this tumor entity, we perform periodical close followup every three months in order to be able to detect and to treat any progression at an early stage by radical surgical intervention. The patient remains alive and well without recurrence of the phyllodes tumor after 18 months.

## 3. Discussion

Phyllodes tumor of the prostate is an uncommon prostatic neoplasm with distinctive clinical and pathological characteristics. It has been assigned numerous names during the last 20 years, including prostatic cystic epithelial-stromal tumor, phyllodes type of atypical hyperplasia, cystadenoleiomyofibroma, prostatic stromal proliferation of uncertain malignant potential, and cystosarcoma phyllodes [1]. The lack of consistency in the nomenclature reflects variability in the histological appearance and the clinical behavior of this tumor. Given the similarities with phyllodes tumor of the breast, the term “phyllodes tumor” seems preferable for this prostatic lesion. The true incidence is unknown, but the condition is rare, with around 80 reported cases.

Although the mean age of patients who present with phyllodes tumors is about 55 years, age range at presentation from 22 to 86 years and 40% of the patients are younger than 50 years [2]. The clinical presentation can mimic that of benign prostatic hyperplasia, with obstruction, dysuria, hematuria, and urinary retention. The obstructive voiding symptoms occur at an age younger than expected for typical prostatic hyperplasia. Additionally, because the tumor often is large, many patients present with a palpable abdominal mass [3, 4]. The prostate is palpably enlarged but soft and may feel spongy. On CT or MRI, phyllodes tumor may appear lobulated and partially cystic. Diagnosis by needle biopsy is difficult because the distinctive architectural features are not well demonstrated. Most cases, like the demonstrated one, are diagnosed initially after transurethral resection, and the prostate gland at transurethral resection is sometimes described as being unusually soft, cystic, or spongy [5].

Histologically, phyllodes tumor of the prostate is biphasic, consisting of stromal and epithelial components arranged to form cysts lined by hyperplastic epithelium. The proliferating stroma is of variable cellularity and sometimes shows subepithelial condensation. The lining epithelium is benign, with basal cell and secretory layers that show immunoreactivity typical of prostatic epithelium, but may show various metaplastic and proliferative changes such as basal cell hyperplasia or squamous metaplasia [1]. There is wide variability in stromal-to-epithelial ratio, stromal cellularity, cytologic atypia, and mitotic activity. These variable features, as well as the presence or absence of necrosis, have been quantified and used to assign a tumor grade. High-grade “malignant” tumors exhibit greater cellularity, mitotic figures, necrosis, and a high stromal-to-epithelial ratio. But the stratification into low, high, and/or even intermediate grades has not been validated. Certainly grade appears to be related to outcome in those patients with an overtly malignant, sarcomatoid component, who are at risk for extensive local pelvic invasion, distant metastasis, and death. Metastatic spread is to lungs, bone, and liver. Lymph node metastases have not been seen. 

Tumor recurrence after transurethral resection is common, occurring in 65% of patients overall, with recurrence rates from 50% for low-grade tumors to 100% for high-grade tumors [5]. Multiple recurrences are often accompanied by progressively increasing biological aggressiveness and sarcomatous transformation. Phyllodes tumors of the prostate are tumors with a definite malignant potential. The use of the term “stromal tumor of uncertain malignant potential” is discouraged, since this term erroneously encompasses and lumps together several unique and distinctive tumors—specifically phyllodes tumor, low-grade stromal sarcoma, and prostatic stromal hyperplasia with atypia. These lesions differ from one another histologically and immunohistochemically, and each requires its own clinical management strategy [6]. Investigations of gene amplifications indicate that epidermal growth factor receptor (EGFR) and androgen receptor are frequently and strongly expressed in both epithelial and stromal components of prostatic phyllodes tumors [7]. Targeted therapy with anti-EGFR and/or antiandrogen agents might be potentially effective for treatment of patients with tumors expressing EGFR and/or androgen receptor in the future.

Since phyllodes tumor is potentially aggressive, complete tumor resection propably provides the best chance for cure. In low-grade tumors alternatively, as in our case, close cystoscopic and transrectal ultrasound surveillance after 2nd transurethral resection without further detectable tumor can achieve local control. However, careful surveillance is essential as progression to higher grades can occur, requiring more aggressive intervention.
